# Characterization of a Recombinant Cathepsin B-Like Cysteine Peptidase from *Diaphorina citri* Kuwayama (Hemiptera: Liviidae): A Putative Target for Control of Citrus Huanglongbing

**DOI:** 10.1371/journal.pone.0145132

**Published:** 2015-12-30

**Authors:** Taíse Fernanda da Silva Ferrara, Vanessa Karine Schneider, Luciano Takeshi Kishi, Adriana Karaoglanovic Carmona, Marcio Fernando Madureira Alves, Jose Belasque-Júnior, José César Rosa, Wayne Brian Hunter, Flávio Henrique-Silva, Andrea Soares-Costa

**Affiliations:** 1 Laboratory of Molecular Biology, Department of Genetics and Evolution, Federal University of São Carlos, São Carlos, SP, Brazil; 2 Department of Biophysics, Federal University of São Paulo, São Paulo, SP, Brazil; 3 Department of Phytopathology and Nematology, University of São Paulo, Piracicaba, São Paulo, SP, Brazil; 4 USDA, ARS, 2001 South Rock Road, Fort Pierce, Florida, United States of America; 5 Protein Chemistry Center and Department of Molecular and Cellular Biology and Pathogenic Bioagents, Ribeirão Preto Medical School, University of São Paulo, Ribeirão Preto, SP, Brazil; Natural Resources Canada, CANADA

## Abstract

Huanglonbing (HLB) is one of the most destructive disease affecting citrus plants. The causal agent is associated with the phloem-limited bacterium *Candidatus* Liberibacter asiaticus (CLas) and the psyllid *Diaphorina citri*, vector of disease, that transmits the bacterium associated with HLB. The control of disease can be achieved by suppressing either the bacterium or the vector. Among the control strategies for HLB disease, one of the widely used consists in controlling the enzymes of the disease vector, *Diaphorina citri*. The insect *Diaphorina citri* belongs to the order Hemiptera, which frequently have cysteine peptidases in the gut. The importance of this class of enzymes led us to search for enzymes in the *D*. *citri* transcriptome for the establishment of alternatives strategies for HLB control. In this study, we reported the identification and characterization of a cathepsin B-like cysteine peptidase from *D*. *citri* (*DCcathB*). *DCcathB* was recombinantly expressed in *Pichia pastoris*, presenting a molecular mass of approximately 50 kDa. The enzyme hydrolyzed the fluorogenic substrate Z-F-R-AMC (*K*_*m*_ = 23.5 μM) and the selective substrate for cathepsin B, Z-R-R-AMC (*K*_*m =*_ 6.13 μM). The recombinant enzyme was inhibited by the cysteine protease inhibitors E64 (IC_50_ = 0.014 μM) and CaneCPI-4 (Ki = 0.05 nM) and by the selective cathepsin B inhibitor CA-074 (IC_50_ = 0.095 nM). RT-qPCR analysis revealed that the expression of the *DCcathB* in nymph and adult was approximately 9-fold greater than in egg. Moreover, the expression of this enzyme in the gut was 175-fold and 3333-fold higher than in the remaining tissues and in the head, respectively, suggesting that *DCcathB* can be a target for HLB control.

## Introduction

Citrus cultivation has considerable worldwide economic importance. Citrus fruits are currently produced in 140 countries, with an annual production of more than 122 million tons. According to the Food and Agriculture Organization of the United Nations, the main citrus producers are China, Brazil, USA, India and Mexico [1]. However, losses occur due to agricultural pests and diseases. Huanglongbing (HLB), also known as citrus greening disease [2, 3], is considered the most serious disease of citrus [4]. HLB has been known in China for nearly hundred years, having first been reported in 1919 [5, 6]. In Brazil (represented by the state of São Paulo) and the United States (represented by the state of Florida), HLB was first reported in 2004 [7, 8, 9] and 2005 [10], respectively. The occurrence of HLB was also confirmed in other countries in North, Central, and South America after the year of 2007 [11, 12, 13, 14].

In Africa, HLB is associated with the bacterium *Candidatus* Liberibacter africanus and the vector is the psyllid *Trioza erytreae* (Del Guercio) (Hemiptera: Triozidae). In Asian and American countries HLB is associated with *Ca*. L. asiaticus and the vector is *Diaphorina citri* Kuwayama (Hemiptera: Liviidae). In Brazil and southern Texas, there is a third variant denominated *Ca*. L. americanus [8, 15], which is less heat tolerant and less prevalent than *Ca*. L. asiaticus [16, 17].

*Candidatus* Liberibacter spp. colonize the conducting vessels of the plant, blocking the phloem and triggering the disease development process. The most common symptoms are blotchy leaf mottle, defoliation, yellow shoots and aborted seeds. The fruit exhibits irregular maturation, inverted coloration, a reduction in size, deformation and frequent dropping [4]. The acquisition of *Ca*. L. asiaticus can occur through either *D*. *citri* nymphs (4^th^ and 5^th^ instars) or adults [18].

If HLB control actions are not adopted, an orchard can become economically unviable in seven to ten years after the onset of symptoms, whereas younger orchards can become economically unviable within five years [19]. Among the control strategies for HLB disease, one of the widely used consists in controlling the disease vector, *Diaphorina citri* Kuwayama through chemical control [20, 21]. The biological control also has been studied. There are two known parasitoids for the control of *D*. *citri*: *Diaphorencyrtus aligarhensis* (Hymenoptera: Encyrtidae) and *Tamarixia radiata* Waterston (Hymenoptera: Eulophidae) [22].

Alternative strategies for insect control have been developed to reduce the dependence on chemical pesticides. There are many reports of transgenic plants overexpressing peptidase inhibitors for insect control, such as sugarcane expressing the soybean Kunitz trypsin inhibitor (SKTI) and soybean Bowman-Birk inhibitor (SBBI), which retard the growth of *Diatraea saccharalis* larvae feeding on the leaves of transformed plants [23]. A 53% mortality rate was found for *Leptinotarsa decemlineata* larvae reared with transgenic potato leaves overexpressing oryzacystatin I [24]. The work of [25] demonstrated that *Myzus persicae* and *Acyrthosiphon pisum* nymphs feeding on *Arabidopsis* plants overexpressing a barley-cystatin presented a significant delay to reach the adult phase, demonstrating the interference of the cystatin in the development of these insects. Another alternative is the development of plants that overexpress double-stranded RNA (dsRNA) to inhibit gene expression on the RNA level. [26] reported the development of transgenic plants overexpressing dsRNA for insect control, describing the expression of 246 bp dsRNA for V-ATPase A in transgenic maize. This strategy led to a significant reduction in the attack of the roots by *Diabrotica virgifera virgifera* LeConte. [27] reported the expression of dsRNA in rice for the midgut genes hexose transporter (*NlHT1*), carboxypeptidase (*Nlcar*) and trypsin-like serine peptidase (*Nltry*) of the hemipteran *Nilaparvata lugens*. The nymphs of this insect that fed on the transgenic rice plants did not exhibit the lethal phenotype, but the transcript level of the target genes was reduced.

*D*. *citri* studies involving RNA interference have been performed to evaluate the effect of gene silencing in the development of the insect, aiming HLB control. Application of a dsRNA specific for five CYP4 genes caused a significant higher mortality in D. citri adults compared to a control group [28]. [29] analyzed EST sequences of *D*. *citri* to identify potential targets for RNA interference in *Bactericerca cockerelli* and suggested that RNAi targets have a potential application against *D*. *citri*, because there is a phylogenetic conservation between the two psyllids. [30] evaluated the effect of topical application of specific dsRNAs for *D*. *citri awd* gene to nymphs and [31] performed the transient expression dsRNA and siRNA for the same gene in the phloem and associated cells of *Citrus macrophylla* and evaluated the effect on insects that fed on the plants. Both works related malformed-wing and reduced survival in adults.

There are many reports about transgenic citrus plants with desirable characteristics [32]. However, few studies based on development of transgenic citrus plants against HLB were performed. [33] reported resistance against HLB of two transgenic sweet orange cultivars ‘Hamlin’ and ‘Valencia’ overexpressing an *Arabidopsis thaliana NPR1* gene constitutively and driven by a phloem specific promoter of *Arabidopsis*. Until the present moment, there is no report of a transgenic citrus plant overexpressing peptidase inhibitors.

For the application of such alternative strategies, targets must been identified and characterized in *Diaphorina citri*. [34] report the identification of cysteine peptidases in the gut of insects belonging to the orders Hemiptera, Diptera and Coleoptera. As *D*. *citri* belongs to the order Hemiptera, it likely has cysteine peptidases in its digestive tract. Given the severity of HLB and the importance of the citrus industry to the global economy, the aims of the present study were to perform the recombinant expression and kinetic characterization of a cathepsin B-like cysteine peptidase (*DCcathB*) from *Diaphorina citri* and analyze the gene expression of *DCcathB* in different insect developmental phases and tissues. These findings can contribute to the validation and possible use of this enzyme as a target for the development of a HLB control strategy.

## Materials and Methods

### *In silico* analysis of *DCcathB* sequence

The search for a gene that encodes a cysteine peptidase in *Diaphorina citri* was performed from transcriptome data developed by the United States Department of Agriculture (USDA) [35]. A partial sequence of a cathepsin B (gi 110456453) was used to search for similarities in the local database through the transcriptome assembly of *D*. *citri* using the BLAST program [36].

*In silico* analysis was performed with a complete sequence of a cathepsin B-like cysteine peptidase using the InterPro database [37] to check the presence of domains for cysteine peptidase and the position of the pro-region. The signal peptide prediction was performed using the SignalP program [38] and potential N-glycosylation sites present in the sequence were analyzed using the NetNGlyc 1.0 Server program [39]. For an accurate analysis of the similarity of *DCcathB* with cathepsin B-like cysteine peptidases from other hemipterans, an alignment was performed using the Multalin program [40].

### RNA isolation and cDNA synthesis

Adults, nymphs and eggs of *D*. *citri* were collected in Fundo de Defesa da Citricultura–Fundecitrus, Araraquara, São Paulo–Brazil, under the responsibility of the Dr. José Belasque Jr. No field studies were performed in the present work. No specific permits were required for the described studies.

Total RNA was extracted separately from each insect phase following the methods described by [41]. cDNA synthesis was performed using the Improm II Reverse Transcription System Kit (Promega) with 2 μg of RNA, 0.5 μg of Oligo dT, 1 U of ImProm II^TM^ reverse transcriptase, in a final volume of 15 μL, following the manufacturer’s instructions. The cycle utilized for reaction was 25°C for 5 min, 42°C for 60 min and 70°C, 15 min. The experiment was performed in thermal cycler (Eppendorf Mastercycler Gradient Thermocycler).

### Construction of expression vector

Full-length cDNA coding for a proenzyme form of a cathepsin B-like cysteine peptidase of *Diaphorina citri* was cloned in the vector pPICZαC in fusion with the secretion α-factor for protein secretion into the medium. The open reading frame coding for the cysteine peptidase protein was obtained through polymerase chain reaction (PCR) amplification using cDNA from adult insects as the template and the specific primers CatBPpicZ-F and CatBPpicZ-R (Table 1) with restriction sites for the enzymes *Eco*RI and *Xba*I. Briefly, 1 μL of template DNA, 200 μM of dNTP (Invitrogen), 2.5 μL of Taq Buffer [100 mM Tris-HCl (pH 8.8 at 25°C), 500 mM of KCl and 0.8% (v/v) of Nonidet P40 (Thermo Scientific)], 1.25 mM of MgCl_2_, 10 pmol of each primer and 1 U of Taq DNA polymerase (Thermo Scientific) were used in a 25 μL reaction. The PCR protocol began at 94°C for 3 min, followed by 35 cycles of 1 min at 94°C, 1 min at 55°C and 1 min at 72°C and final extension for 7 min at 72°C in a thermal cycler (Eppendorf Mastercycler Gradient Thermocycler). The amplification product was purified, digested with *Eco*RI and *Xba*I and inserted into a pPICZαC vector (Invitrogen) previously digested with the same enzymes. The ligation reaction between the amplicon and the pPICZαC vector was performed in a standard reaction using T4 DNA Ligase (Invitrogen) and transformed in *E*. *coli* DH5α competent cells using the calcium chloride method [42].

**Table 1:** Oligonucleotide primers used in this work.

Name	Sequence 5’- 3’
CatBPpicZ-F	ATCGGAATTCTACGTTCCACAGAGACTGGAC
CatBPpicZ-R	AGCTTCTAGATTAAACTTGCAAAAACTGTGGGT
CatB_Int-F	AGCCAACTCTAAACAGGC
CatB_Int-R	ATGTGAGCTTTCTTGCCC
α–factor F	TACTATTGCCAGCATTGCTGC
*AOX1* –R	GCAAATGGCATTCTGACATCC
CathBD.citriRT_F	CAGCACAACTTCGGTGATT
CathBD.citriRT_R	CAATGGTCGTTCCAAGAGT
ActinRT_F	ACCATCGGAAACGAAAGAT
ActinRT_R	CGTGGATACCGCAAGATT
18S_Foward	TTGTCCTCAAGAAGGCTGA
18S_Reverse	TTGTATTGTCTGGGGTTGG

Colonies were screened on LB low salt agar medium (Sigma Aldrich) plates with the addition of 25 μg/mL of Zeocin™. The recombinant clone pPICZαC_*DCcathB* was selected and sequenced using the dideoxy method [43] in the MegaBaceTM1000 DNA sequencer using the DYEnamic ET Terminator kit (GE Healthcare). The sequencing primers were CatB_Int-F, CatB_Int-R and the universal primers were α-Factor (forward) and *AOX1* (reverse) (Table 1).

### Recombinant expression of *DCcathB* in *Pichia pastoris*

The recombinant plasmid was linearized with the enzyme *Pme*I (New England Biolabs) and transformed into *Pichia pastoris* KM71H strain competent cells through electroporation. The competent yeast cells were prepared following the method described [44]. The transformation was performed using the Gene Pulser Unit (BIO-RAD) in a cuvette (0.2 cm, 25 uF, 200 Ω), following the instructions of the EasySelect *Pichia* Expression Kit (Invitrogen). The culture was plated on YPDS medium (1% yeast extract, 2% peptone, 2% dextrose, 1 M of sorbitol and 1.5% bacteriological agar) containing 100 μg/mL of Zeocin™ and additional plates with 200 μg/mL of Zeocin™ for the selection of multi-copy clones. The plates were incubated for 48 h at 30°C. PCR was performed with transformed yeast colonies to screen the recombinant clones [45].

The colonies selected by PCR were screened for protein expression in 24-well plates with 3 mL of BMGY medium (1% yeast extract, 2% peptone, 100 mM of potassium phosphate buffer (pH 7.0), 1.34% yeast nitrogen base, 4 x 10^−5^% biotin and 1% glycerol) for 48 h at 30°C and 250 rpm. The cells were then centrifuged for 5 min at 1700 x *g* and re-suspended in 2 mL of BMMY medium (same composition as the BMGY medium, but the replacement of glycerol with 0.5% methanol) for expression for 144 h at 30°C and 250 rpm, with the daily addition of methanol (0.75%). Aliquots of 100 μL were removed every 24 h and 10 μL of the culture supernatant was analyzed using SDS-PAGE 12% [46] stained with Coomassie Blue R-250. For large scale expression, a colony with the highest level of expression was selected and inoculated in 10 mL of BMGY for growth for 24 h at 30°C and 250 rpm. The culture was transferred to a flask with 500 mL of BMGY and incubated at 30°C and 250 rpm until OD_600_ 4–5. The cells were centrifuged for 5 min at 1500 x *g* and homogenized in 100 mL of BMMY for 24 h at 30°C and 250 rpm. The culture was centrifuged and the supernatant containing the secreted recombinant protein was vacuum filtered through a 0.44 μm PVDF membrane.

### Purification of recombinant protein *DCcathB* by affinity chromatography

The yeast medium containing the secreted *DCcathB* protein was purified through an affinity chromatography in a Ni-NTA superflow nickel column (Qiagen). The column was previously equilibrated with buffer containing 10 mM Tris-HCl, 100 mM NaCl and 50 mM NaH_2_PO_4_, pH 8.0. All purification steps were performed at 4°C. The protein was eluted with the same buffer with increasing imidazole concentrations (10, 25, 50, 75, 100 and 250 mM). The purified protein was analyzed in 12% SDS-PAGE. Fractions containing the purified protein were dialyzed using membranes of 3500 MW (Pierce) for 2 hours at 4°C in buffer containing 10 mM Tris-HCl, 100 mM NaCl and 50 mM NaH_2_PO_4_, pH 8.0. The protein was then concentrated in the SPD1010 SpeedVac^®^ System (ThermoSavant) for 3 hours and in Vivaspin™ 3000 MWCO (GE Healthcare) for 1.5 h. The protein concentration was determined using Bradford’s method [47] in Hitachi U-5100 spectrophotometer.

### Mass spectrometry analysis of *DCcathB*

Protein samples were separated by SDS-PAGE 12% and analyzed in a MALDI TOF/TOF mass spectrometer after in-gel trypsin digestion. Briefly, selected gel bands were excised and combined. SDS and CBB were removed by washing the gels three times with 50% ACN in 0.1M ammonium bicarbonate (pH 7.8), followed by dehydration in neat acetonitrile. Gel bands were dried in a Speed Vac instrument (Savant, New York, NY) and were swollen in 20 μL of 0.1 M (pH 7.8) ammonium bicarbonate containing 0.5 μg trypsin (Promega, Madison, USA), followed by the addition of 50 μL of 0.1 M ammonium bicarbonate to cover the entire gel piece. Trypsin hydrolysis was carried out at 37°C for 24 h and the reaction was stopped by the addition of 5 μL of formic acid (98%). Peptides were extracted from gel pieces and desalted in microtips filled with POROS R2 (PerSeptive Biosystems, Foster City, CA) previously equilibrated in in 0.2% formic acid. After loading, the sample was desalted by washing two times with 150 μL of 0.2% formic acid. The peptides were eluted from the microtips with 30 μL of 60% methanol/5% formic acid. The sample was dried down in a speed vac and mixed with matrix solution (5 mg/mL of α-cyano-4 hydroxycinnamic acid in 50% acetonitrile/0.1% trifluoroacetic acid) and applied to the MALDI target plate as air dried drops at room temperature. The MALDI TOF/TOF mass spectrometer (Axima Performance, Kratos-Shimadzu, Manchester, UK) was calibrated with a mixture of bradykynin fragment (1–7), angiotensin II, renin and ACTH (mass accuracy < 50 ppm). The CID-MS/MS spectra of each detected ion were obtained in data-dependent acquisition mode. The peak list was obtained from CID-MS/MS spectra using Launchpad v. 2.8 (Kratos-Shimadzu, Manchester, UK) and submitted to a database search using the MASCOT program version 2.2.04 (Matrixscience, Manchester, UK) directly against the ORF sequence. The database search parameters accepted one missing trypsin cleavage and methionine oxidation. Mass tolerance was 1.2 Da for precursor ions and 0.8 Da for product ions. Protein was considered to be identified by MASCOT score > 56.5% level of significance (p < 0.05) and FDR less than 1%. The amino acid sequences of the tryptic peptides were compared with the amino acid sequence of the cysteine peptidase in the USDA database of the *D*. *citri* transcriptome and the amino acid sequence obtained by sequencing performed at the Molecular Biology Laboratory of the Federal University of São Carlos, SP, Brazil.

### Kinetic characterization of *DCcathB*

According to [48], different recombinant systems have been used for cathepsin B expression but in all of these the peptidases were activated following processing. Furthermore, according to [49], to generate active peptidases it is necessary to remove the propeptide region and this can be removed autocatalytically by incubating the protein in acetate buffer at pH 4–4.5. The activation of the recombinant enzyme was performed using the method described by [49] with modifications. The enzyme in buffer containing 10 mM Tris HCl, 50 mM NaH_2_PO_3_ and 100 mM NaCl at pH 8.0 was activated with the addition of 50% acetic acid until reaching pH 5.0 and incubation for 60 min at 37°C.

Assays for determination of the catalytic activity of *DCcathB* cysteine peptidase (2.14 nM) were performed in 100 mM sodium acetate buffer (pH 5.5) containing 2.5 mM DTT (dithiotreitol) in final volume of 500 μL. The enzyme was pre-activated for 3 min at 30°C before the addition of the substrate. The fluorogenic substrates Benzyloxycarbonyl-L-phenylalanyl-L-arginine-4-methylcoumaryl-7-amide (Z-F-R-AMC) (Calbiochem) and Benzyloxycarbonyl-L-arginyl-L-arginine-4-methylcoumaryl-7-amide (Z-R-R-AMC) (Calbiochem) were added at concentrations ranging from 1 to 75 mM and the enzyme activity was continuously monitored in a Hitachi F-2500 spectrofluorometer with fluorescence measured at λ_ex_ = 380 and λ_em_ = 460 nm following the procedure described by [50]. The Michaelis-Menten constant (*K*_*m*_) was determined using the GraFit program [51]. Tests were carried out in triplicate.

### Inhibition assays of *DCcathB* activity

The inhibition *DCcathB* activity was determined spectrofluorometrically using the substrate Z-F-R-AMC. Fluorescence was measured in a Hitachi F-2500 spectrofluorometer at λ_ex_ = 380 and λ_em_ = 460 nm. Inhibitory activity was determined by measuring the residual hydrolytic activity of the cysteine peptidase. The enzyme (2,14 μM) was added to 100 mM sodium acetate buffer (pH 5.5) containing 2.5 mM DTT, in a final volume of 500 μL, and pre-incubated for 3 minutes at 30°C. Then, the substrate Z-F-R-AMC (37.5 mM) was added and the *DCcathB* activity was measured in the presence of the following inhibitors: CaneCPI-4 (0,04; 0,08; 0,12; 0,16; 0,20 nM) [52], synthetic epoxide peptide E-64 (L-trans-Epoxysuccinyl-leucylamido(4-guanidino)butane) (Calbiochem) (0,004, 0,008; 0,012; 0,016; 0,020 μM) [53] and synthetic inhibitor CA-074 [N-(l-3-trans-propylcarbamoyloxirane-2-carbonyl)-L-isoleucyl-L-proline] (Calbiochem) (0,026; 0,052; 0,078; 0,104; 0,13 nM) [54]. The residual *DCcathB* activity was measured. The inhibition constant (*K*_*i*_) was calculated following Morrison’s procedure using the GraFit program. All experiments were carried out in triplicate. The results were used to determine the IC_50_ [55] for E-64 and CA-074.

### Gene expression analysis of *DCcathB* in insect developmental phases and tissues

The developmental phases of egg, nymph (5^th^ instar) and adult and the tissues of head, gut and remaining tissues were used for *DCcathB* gene expression analysis by reverse transcription quantitative PCR (RT-qPCR). Egg, gut, head and remaining tissues were collected with a needle, placed in a 1.5-mL tube with 500 μL of Trizol reagent (Invitrogen) and incubated on ice to maintain the integrity of the material. The RNA isolation was performed according to [41]. RNA was quantified in a Nanodrop Spectrophotometer ND-1000 (Thermo Scientific) and integrity was checked in 1% agarose gel stained with ethidium bromide.

Each RNA sample was individually treated with 1U/μL of DNase I Amplification Grade (Invitrogen) and 10x DNase I Reaction Buffer (final concentration 1x). The samples were incubated for 15 min at room temperature to remove traces of genomic DNA. DNase I was inactivated by adding of 1 μL of 25mM EDTA/μL of DNase and incubation at 65°C for 10 min.

The primers CathBD.citriRT_F, CathBD.citriRT_R, ActinRT_F, ActinRT_R, 18S_Foward and 18S_Reverse (Table 1) were designed with the Primer 3 program, version 4.0 [56]. To ensure that the *DCcathB* primers designed to RT-qPCR will be specific and amplify only the fragment of interest of this enzyme, we used the Primer-BLAST Program [57] and an alignment with the primers with cathepsins B-like identified in the *D*. *citri* transcriptome [35] was performed.

The amplification reactions were conducted using 5 μL of 1x Platinum SYBR Green qPCR Supermix UDG (Invitrogen), 1 μL of cDNA (100 ng) and 0.4 μM of each primer, in a final volume of 10 μL. Due to the constitutive expression, the reference gene chosen for analysis of *DCcathB* gene expression in developmental phases was the actin gene (genbank accession number: 110456519) and for *DCcathB* gene expression analysis in tissues the reference gene was 18S rRNA (genebank accession number: 110671475). Both genes are cited as reference in studies involving expression analyses in *D*. *citri* [28, 30, 58, 59]. The reactions were conducted in triplicate in the Eco Real-time System (Illumina) using the following reaction cycle: 50°C for 2 min, 95°C for 2 min, followed by 40 cycles of 95°C for 30 s, 54°C for 30 s and 72°C for 40 s. The melt curve was performed using the following cycle: 95°C for 15 s, 54°C for 15 s and 95°C for 15 s, to analyze the absence of non-specific amplification products.

The data were analyzed using the 2^-ΔΔCt^ method [60]. The standard curve was performed using a serial dilution of cDNA with the initial concentration of 100 ng. No template controls (NTC) were performed to ensure the absence of sample contamination in every run. The following parameters were established for the reactions: efficiency > 90%, slops > -3.25 < -3.6 and R2 > 98%. The Relative Expression Software Tool (REST) 2005 version 1.9.12 from Qiagen [61] was used to determine the significance of the values obtained in the gene expression analysis and the difference in gene expression among phases and tissues was considered significant when p-value was less than 0.05 with a 95% confidence interval.

## Results and Discussion

### *DCcathB* sequence analysis

A complete cDNA sequence encoding the *D*. *citri* cathepsin B-like cysteine peptidase, *DCcathB*, with open reading frame of 1125 bp (genbank accession number (KT835051) was identified using the databank of the *Diaphorina citri* transcriptome [35]. The protein has a predicted sequence of 374 amino acids and a relative molecular mass of 41.9 kDa. According to the analysis performed in the InterPro database, *DCcathB* presented characteristics of a cysteine peptidase with regions that potentially corresponds to an active site of cathepsins B represented by the amino acid residues at the positions 142–153 and 315–325. The *DCcathB* pro-region of 38 amino acids (DIVDQVNNNVTSTWQARHNFHPDTPVSYLSSLAGTRPL) extends from residues 32 to 69 and the mature peptide extends from residues 70 to 374. The signal peptide of 17 amino acids, (MWAVGVLVLIATQSLAI) as predicted by the Signal P Program and four potential N-glycosylation sites were predicted using the NetNGlyc 1.0 Server.

**Fig 1:**
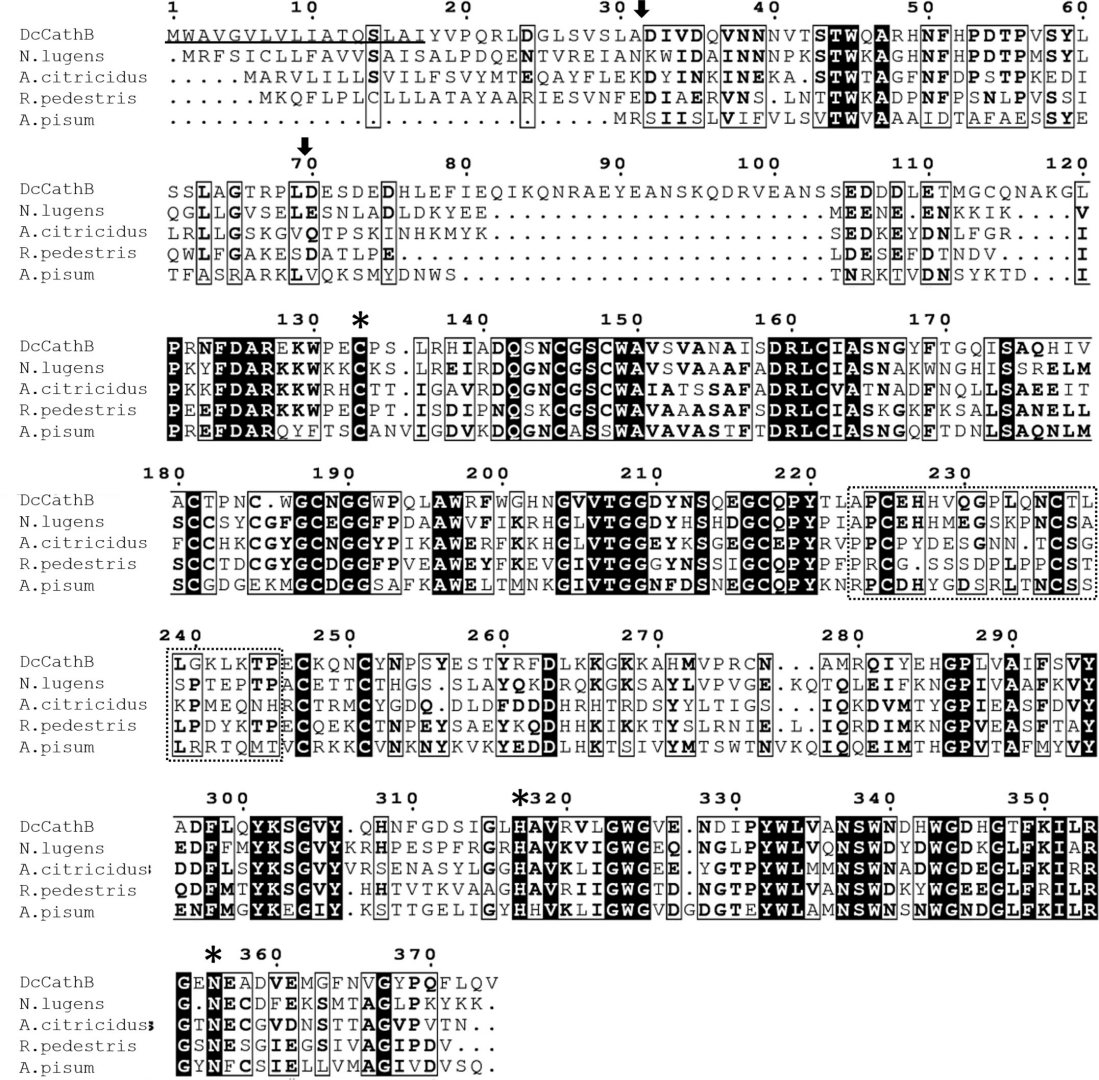
Alignment of *DCcathB* with similar cathepsin B-like cysteine peptidases from hemipterans. *Diaphorina citri* (genbank accession number: 110456454), *Nilaparvata lugens* (genbank accession number: 22535408), *Aphis citricidus* (genbank accession number: 161343879), *Riptortus pedestris* (genbank accession number: 501291537), *Acyrthosiphon pisum* (genbank accession number: 209863079). Conserved identical residues are marked in black boxes and white boxes show conserved residues with more than 50% identity. The *DCcathB* predicted peptide signal of seventeen residues is underlined in black. The probable occlusion loop characteristic of cathepsin B-like cysteine peptidases is represented by the dashed box. The potential cleavage site between the propeptide (residues 32 and 69) and mature *DCcathB* is indicated by an arrow. The predicted conserved catalytic triad C-H-N is indicated by asterisks. Alignment was generated using the Multalign program with default parameters.

The features of *DCcathB* were displays in Fig 1. The alignment shows the presence of the region corresponding to the occlusion loop, which is characteristic of cathepsin B-like enzymes [62]. The cleavage site between the pro-peptide and mature protein is predicted to occur in the sequence PL^DESD…, resulting in D^70^ as the N-terminal amino acid of the mature enzyme. The sequence of pro-peptide region contains a unique N-glycosylation site at N^40^ and the sequence correspondent to the mature protein contains three N-glycosylation sites at N^102^, N^235^ and N^253^. The papain cysteine peptidase family has a pro-region that performs functions such as transport to endosomal or lysosomal compartments for the correct folding of the active mature protein and pro-enzyme activity inhibition [49].

### *DCcathB* expression and purification

**Fig 2:**
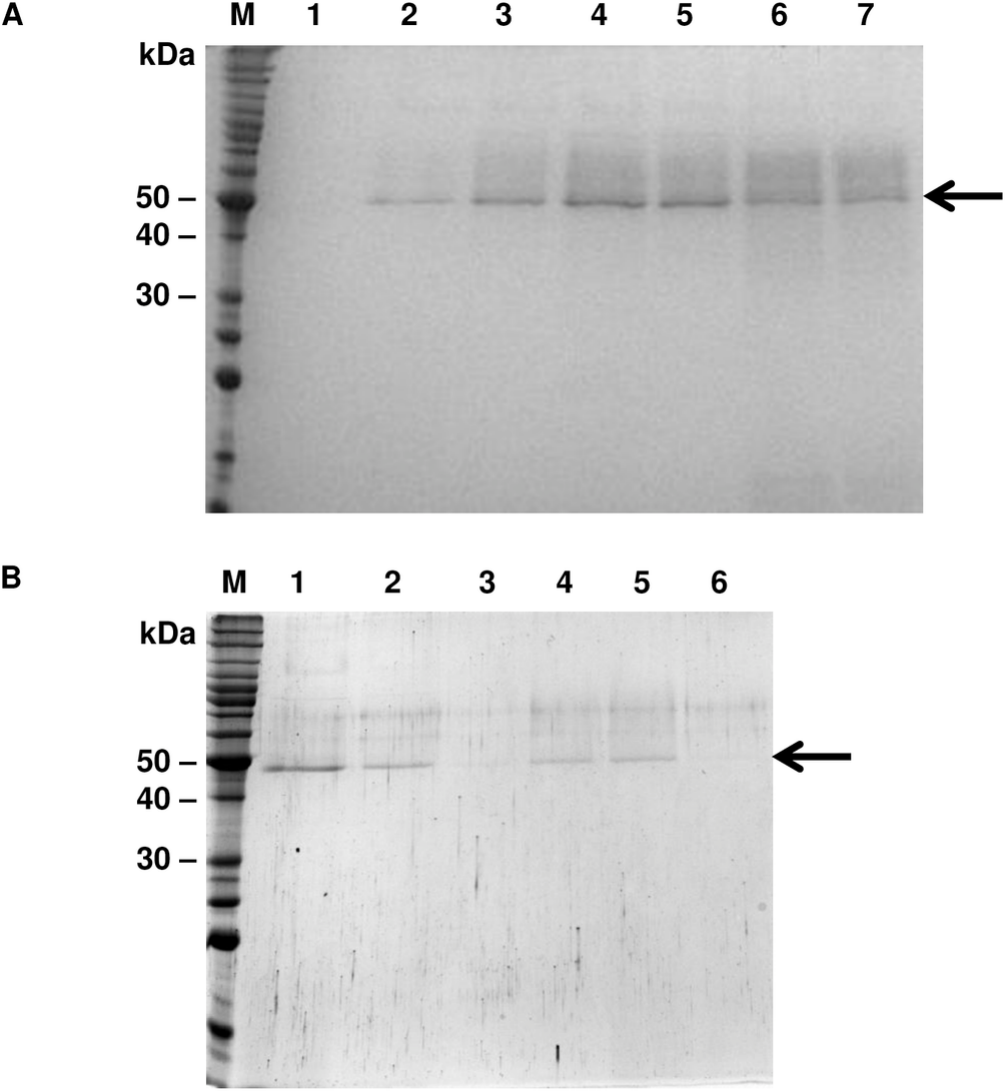
*DCcathB* expression. A–SDS-PAGE 12% stained with Coomassie blue showing the induction of pPICZαC_*DCcathB* supplemented with 0.75% methanol. M: molecular weight BenchMark™ Protein Ladder (Invitrogen). Induction Times: 1–0 h; 2–24 h; 3–48 h; 4–72 h; 5–96 h; 6–120 h; 7–144 h. B–Purification of *DCcathB*. M: molecular weight marker (Invitrogen). 1—Eluate. 2—Wash buffer without imidazole. 3—Protein eluted in 10 mM imidazole. 4—Protein eluted in 10 mM imidazole. 5—Protein eluted in 25 mM imidazole. 6—Protein eluted in 25 mM imidazole.

The SDS-PAGE 12% analysis revealed the expression of the recombinant protein at 24 hours of induction (Fig 2A) and the purified *DCcathB* as a single and intact band of approximately 50 kDa corresponding to the protein eluted in imidazole fractions of 10 and 25 mM (Fig 2B). The yield of purified *DCcathB* protein was 2 mg/L of *P*. *pastoris* cell culture.

The predicted molecular mass of *DCcathB* is 41.9 kDa. However, due to the fusion with α-factor, c-*myc* epitope and C-terminal histidine tag, the expected size for the recombinant protein is approximately 46 kDa. SDS-PAGE analysis revealed a band of 50 kDa probably corresponding to the glycosylated protein.

### Mass spectrometry analysis

**Fig 3:**
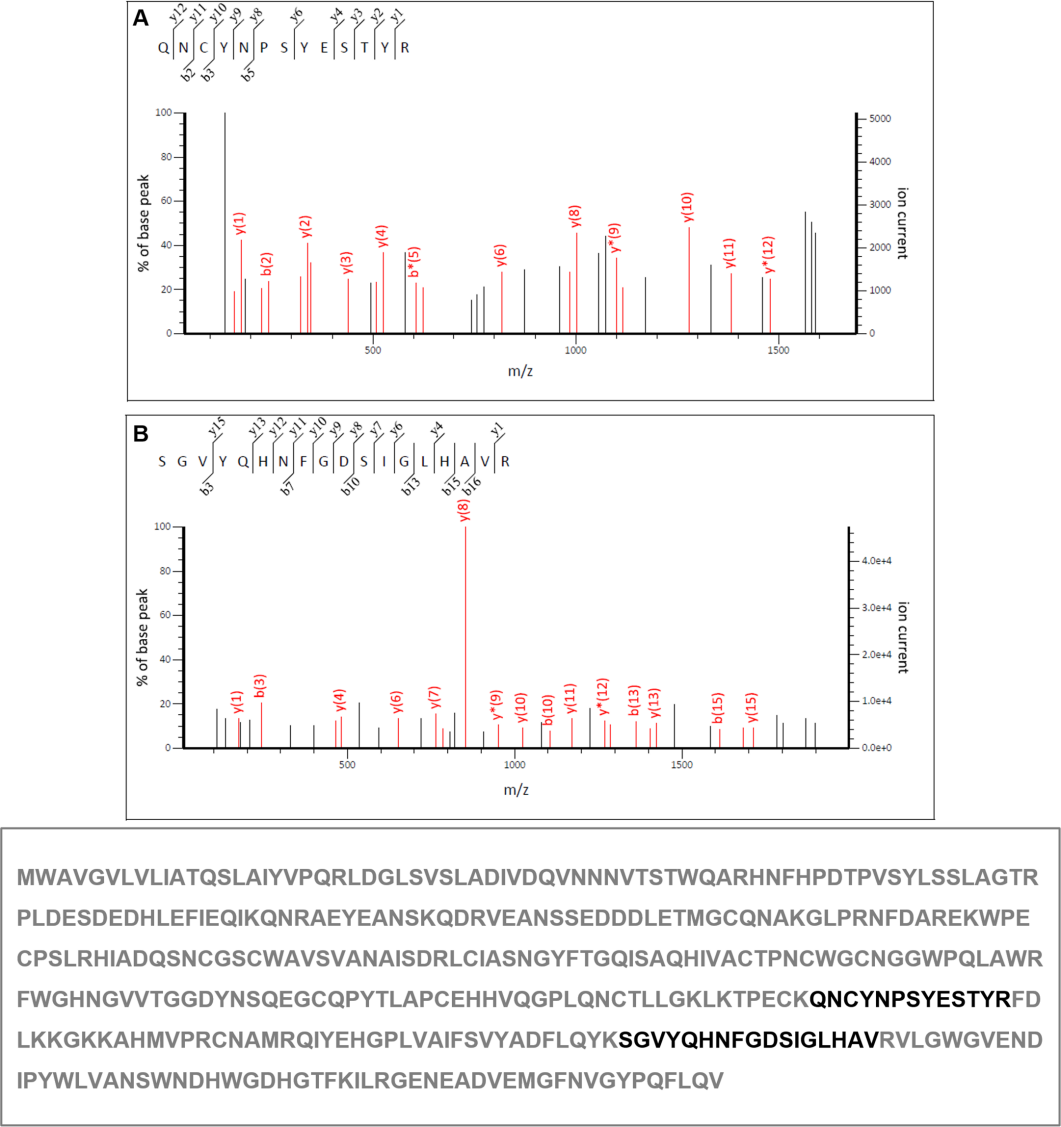
CID-MS/MS spectra of tryptic peptides from *DCCathB*. Amino acid sequences were deduced from product ions and identified (bold) in the cysteine peptidase sequence deduced from *D*. *citri* ESTs.

For the verification of the *DCcathB* amino acid sequence, the purified protein was subjected to sequencing by mass spectrometry. The MALDI TOF/TOF data obtained by CID-MS/MS provided the amino acid sequences of two peptides: QNCYNPSYESTYR (m/z 1625.07 ± 0.41 at residue position 249 to 261) (Fig 3A) and SGVYQHNFGDSIGLHAVR (m/z 1957.32 ± 0.38 at residue position 303 to 320) (Fig 3B). These tryptic peptides correspond to 8.3% sequence coverage and a total MASCOT score of 185. The results obtained by mass spectrometry identified two peptides present in the amino acid sequence of the predicted protein obtained in the USDA database of the *D*. *citri* transcriptome (lcl|Sequence 1 ORF: 50.1174 Frame +2 Cathepsin B) as well in the sequence generated by the sequencing of the pPICZαC_*DCcathB* expression plasmid. The results obtained by mass spectrometry confirm the identity of the purified protein as a cathepsin B-like cysteine peptidase.

### Determination of enzyme activity

As the purified *DCcathB* was produced in its pro-enzyme form, a strategy was adopted to remove the pro-region for subsequent enzyme activation. Once activated, autocatalytic cleavage occurs and the mature protein can process other precursor molecules, with the occurrence of a chain reaction that allows the removal of the pro-region [63].

The *DCcathB* enzyme was able to hydrolyze the substrates Z-F-R-AMC and Z-R-R-AMC substrates and *K*_*m*_ values were determined. For Z-F-R-AMC the *K*_*m*_ value was 23.5 μM. This value is very similar to the *K*_*m*_ of the cathepsin B-like cysteine peptidases yEmCBP1 and yEmCBP2 from *Echinococcus multilocularis* (*K*_*m*_ = 20.45 and 27.17 μM, respectively) [64]. For Z-R-R-AMC the *K*_*m*_ value was 6.13 μM, demonstrating greater affinity. Specificity analysis of cathepsin L midgut *Tenebrio molitor* was performed using different substrates, demonstrating capability to hydrolyze the substrate Z-R-R-AMC, but a greater preference for substrates with hydrophobic residues at the P2 position substrate [65]. However, cathepsin B unlike other cysteine peptidases from papain family, can accept substrates with arginine residue at the P2 position due to the location of an E^245^ residue in the subsite S2. This residue is important for the specificity of cathepsin B [66]. This result is important for the characterization of *DCcathB* therefore demonstrated the enzyme is capable of hydrolyzing the Z-R-R-AMC substrate, a selective substrate for cathepsin B.

### *DCcathB* catalytic activity inhibition

The inhibitory activity of *DCcathB* was evaluated using the sugarcane recombinant cystatin CaneCPI-4 [52] and the synthetic inhibitors CA-074 and E-64. CaneCPI-4 efficiently inhibited *DCcathB* catalytic activity, with a *K*_*i*_ value of 0.05 nM, which demonstrated a strong interaction between the *D*. *citri* cathepsin B-like cysteine peptidase and the cystatin. This value is consistent with data described by [67], who report that the recombinant human cystatin C inhibits human cathepsin B activity, with a *K*_*i*_ value of 0.38 nM. Furthermore, [52, 68] respectively obtained *K*_*i*_ values of 0.5 nM and 0.83 nM for CaneCPI-4 against recombinant human cathepsin B. Cystatins are competitive and reversible inhibitors of cysteine peptidases [69]. The structure of cathepsin B-like cysteine peptidases has an occluding loop that blocks the cleft of the enzyme active site [62] and hinders the access of cystatin to this site, resulting in high *K*_*i*_ values for most cystatins against cathepsin B when compared to other cysteine peptidases that lack this loop [70, 71, 72].

Structural differences between the cystatin CaneCPI-4 and other cystatins may be the key to the inhibitory profile against cathepsin B. This cystatin is included in group III of the cystatin classification and lacks the N-terminal exclusive phytocystatin consensus sequence LAR-[FY]-N-[VI]-x(3)-N motif. Moreover, the conserved motif is represented by QVVAG in the cystatin superfamily, but is represented by QVVSG in CaneCPI-4 [52, 73]. [68] conducted modeling studies by homology to determine regions of phytocystatins responsible for cathepsin B catalytic activity inhibition. Analyzing CaneCPI-4, variations were found in some hydrophobic amino acids between the α-helix and the β sheet, including a glutamine residue at position 30 and glycine at positions 47 and 56, which accounted for a decrease in hydrophobic interactions in the hydrophobic core. These changes contribute to flexibility and, consequently, inhibitory activity against human cathepsin B. The inhibition assays performed with CaneCPI-4 and *DCcathB* clearly demonstrate the high affinity of this inhibitor for the *DCcathB* enzyme.

Analyzes of *DCcathB* inhibition activity using the inhibitors E-64 and CA-074 were also performed and demonstrated an interaction between the enzyme and both inhibitors. The IC_50_ was 0.095 nM for CA-074 and 0.014 μM for E-64. CA-074 is a selective inhibitor of cathepsin B [54]. The interaction between CA-074 and *DCcathB* enzyme is evidence that *DCcathB* is *a* cathepsin B like enzyme.

### *DCcathB* gene expression in insect developmental phases and tissues

Studies with cathepsin B-like cysteine peptidases in insects indicated that these enzymes have a role in several biological processes as digestion of food proteins in midgut [34, 74, 75]; degradation and mobilization of yolk proteins during embryogenesis [76, 77, 78, 79]; programmed cell death; metamorphosis [80, 81, 82] and defense against enemies [83, 84]. A detailed study about the differential expression between infected and non-infected *D*.*citri* nymphs and adults demonstrated that a cathepsin B-like is down-regulated and categorized in defense/immune response-related contigs [84].

**Fig 4:**
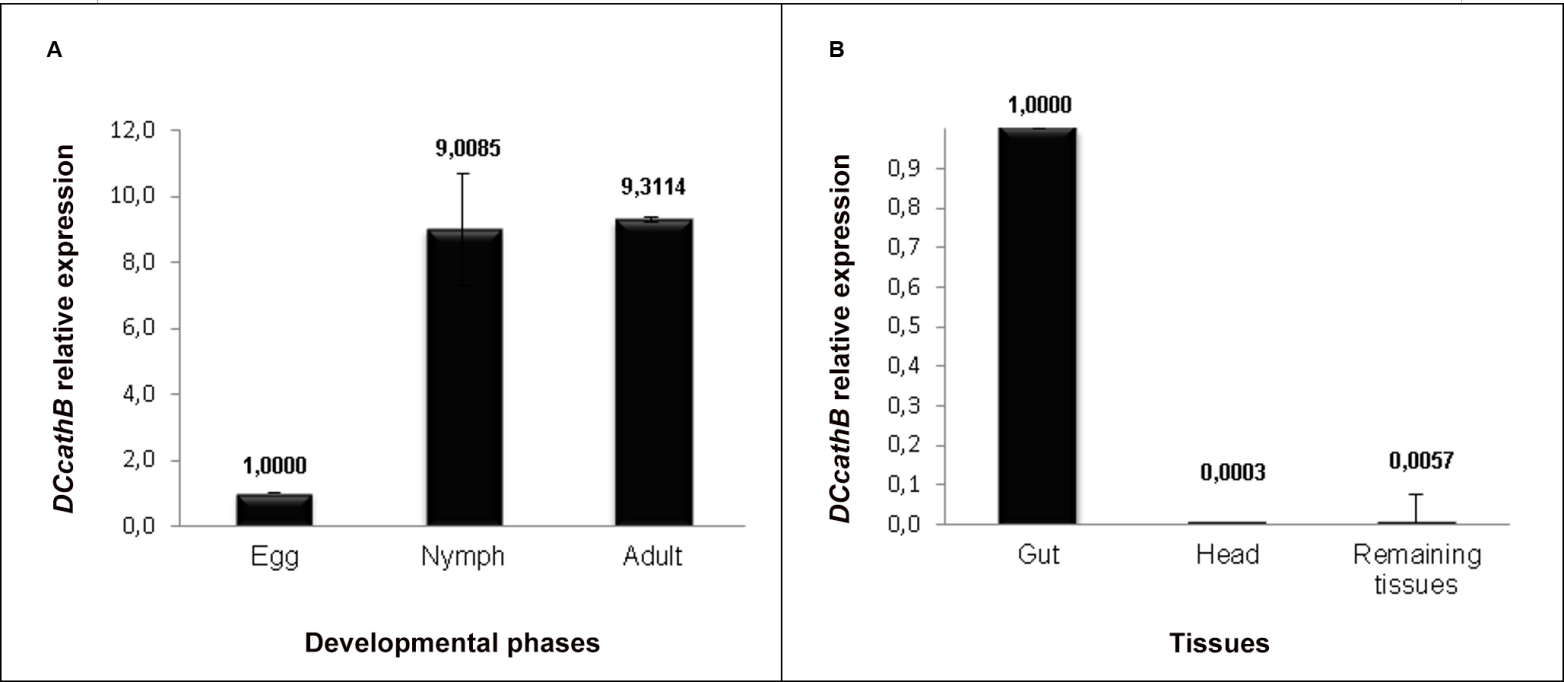
*DCcathB* expression analysis by RT-qPCR in *D*. *citri* egg, nymph and adult (A) and head, gut and remaining tissues (B). The quantification calibrator in developmental phases was egg and for the tissues was gut. Error bars were calculated according [60]. The difference was significant when the p-value was lower than 0.05.

For better understanding the role of *DCcathB* in *D*. *citri*, RT-qPCR was performed to analyze the gene expression of the *DCcathB* in developmental phases of *D*.*citri*: egg, nymphs (5^th^ instar) and adults and in the following tissues: head, gut and remaining tissues. Fig 4A shows the gene expression pattern of *DCcathB* in egg, nymph and adult phases. *DCcathB* presented lower expression in egg, suggesting that *DCcathB* has a basal level of expression in this phase. [85] performed the gene expression analysis of a digestive cathepsin L-like of *Sphenophorus levis* and described a basal expression of the enzyme at the egg. However there was a notable increase in the production of mRNA encoding *DCcathB* in *D*. *citri* nymphs 9-fold and adults 9.3-fold in comparison to the egg phase. This result is interesting because the nymph phase is very important for the acquisition and transmission of the CLas. Moreover, when nymphs fed on CLas-infected plants, the concentration of the bacterium increased significantly and the transmission to the plants is more effective. The adults infected in the nymph phase are able to transmit the bacterium immediately after emergence. In contrast, adults that fed on infected plants did not presented significantly increase of the concentration and are poor transmitters of the bacterium [84, 86, 87]. Thus, a strategy that uses *DCcathB* as target for *D*. *citri* nymph control may be effective.

Gene expression analysis of *DCcathB* in *D*. *citri* head, gut and remaining tissues showed notable increase in the production of mRNA encoding *DCcathB* in the gut, being 3333-fold and 175-fold, respectively, relative to the head and remaining tissues. Fig 4B shows gene expression pattern of *DCcathB* in each tissue analyzed. This result suggests that this enzyme has a digestive function in *D*. *citri*. Several studies reported the presence of cathepsins B in the gut of Hemipterans, demonstrating the importance of these enzymes in digestion process of this insect class. In other Hemiptera insect, the aphid *Acyrthosiphon pisum*, 28 cathepsins B-like were identified and five cathepsins B-like were preferentially expressed in the gut [88]. The presence of cathepsins B-like that are specifically expressed in the gut of *Tuberaphis styraci*, *Nilaparvata lugens*, *Rhodnius prolixus* and *Triatoma infestans* was reported by [83, 89, 90, 91].

Considering that *DCcathB* is highly and preferentially expressed in the gut and nymph phase, our results contribute to the establishment of future strategies of control possibly based on the overexpression of peptidase inhibitors in citrus plants for HLB control.
